# Open defecation and attainment of Sustainable Development Goal Six: evidence from Kintampo Surveillance System, Ghana

**DOI:** 10.4314/gmj.v55i4.7

**Published:** 2021-12

**Authors:** Sulemana W Abubakari, Felix B Oppong, Kenneth Wiru, Grace Manu, Edward A Apraku, Mahama Abukari, Charles Zandoh, Kwaku P Asante

**Affiliations:** Kintampo Health Research Centre, Research and Development Division, Ghana Health Service, Kintampo, Ghana

**Keywords:** Open-defecation, Sustainable Development Goals, Health and Demographic Surveillance System, Kintampo

## Abstract

**Objective:**

This study examined whether the open-defecation (OD) free target is achievable by 2030.

**Design:**

Longitudinal study

**Setting:**

Seven sub-Districts of Kintampo North Municipal, and five sub-Districts of Kintampo South District

**Data source:**

Kintampo health and demographic surveillance system

**Participants:**

Data was collected from household heads or their representatives over a 12-year period from 2005 to 2016.

**Main outcome:**

Open-defecation and attainment of OD free by 2030

**Results:**

In an exploratory analysis, the correlation between the total number of households, year, and total number of OD households was obtained. The average percentage yearly increase or decrease in OD was computed and used to project the percentage of OD for the years 2020, 2025 and 2030. In addition, geo-spatial technology was used to visualize variability in OD across the twelve sub-Districts. The results showed that the OD free target is not achievable in 2030 or even if the current trend continues. In 2016, 44.2 per cent of the 31,571 households defecated openly. In six out of the 12 sub-Districts, more than half of the households openly defecated. Four out of these six sub-Districts were in the Kintampo North Municipality.

**Conclusion:**

The 2030 OD free target is not achievable in the Kintampo districts of Ghana if the current trend continues.

**Funding:**

Kintampo Health Research Centre funded this work

## Introduction

As part of the Sustainable Development Goals (SDGs), target 6.2 of SDG 6 aims at ending open defecation (OD) globally by 2030.^1^, ^2^ However, progress to date has been very slow, especially for countries in sub-Saharan Africa (SSA).^3^ Globally, estimates show that close to 1 billion people of the world's population still practice OD, and about 842,000 people die annually from sanitation-related diseases.^4^–^6^ In SSA alone, about a quarter of a billion (220 million) people are involved in OD.^4^ It is estimated that three out of five Ghanaians practice OD and that in the last 25 years, Ghana made only one per cent progress at eliminating the practice of OD.^7^

No country in SSA achieved the Millennium Development Goals (MDGs) Target 7C that aimed to “halve, by 2015, the proportion of people without access to safe drinking water and basic sanitation”^8^.

However, a few countries, namely Angola, Botswana, South Africa and Ethiopia, made some progress.^5^,^9^,^10^ Galan et al, 2013 estimated how many countries would achieve OD free status by 2015. They observed that only a few countries in sub-Saharan Africa made significant progress toward reducing OD prevalence. Also, they noted that only one country out of 34 countries analyzed, Angola, is expected to end OD by 2015.^11^

Poor hygiene and sanitation practices expose the population to avoidable diseases such as transmissible infectious diseases, diarrhoea, typhoid and cholera, and viral infections that tremendously affect productivity.^11^ OD increases the spread of diarrheal illnesses, which are one of the leading causes of morbidity and mortality among children less than five years old in SSA.^12^,^13^

There is the need to monitor SDGs and measure whether the targets are being met or not. Such monitoring and evaluation are essential for ensuring the achievement of the targets that have been set. However, low and middle-income countries cannot do this and often miss such targets. The Kintampo Health and Demographic Surveillance System (KHDSS) offers the opportunity for such monitoring and evaluation. Therefore, this paper monitors target 6.2 of SDG 6 by reporting on whether OD is achievable or not in the Kintampo districts of Ghana.

## Methods

### Study area and population

Households' data for this study was extracted from the KHDSS, which covers the Kintampo North Municipal and the Kintampo South District located in the middle part of Ghana. The districts are mainly rural, and their capitals, Kintampo and Jema, are semi-urban.^14^,^15^ The districts had an estimated population of about 163,816 as at December 2019.^16^

The KHDSS routinely updates the entire population by collecting data on births, deaths and migrations, and other demographic and health information. The study population for this study comprised household heads or their representatives that were residents in the KHDSS area from January 1^st^ 2005 to December 31^st^ 2016 as shown in Table 1.

**Table 1:** Yearly prevalence of open-defecation households and yearly percentage increase in open-defecation households in Kintampo North Municipal and Kintampo South District

Year	Number of households	Number of open defecation households	% of open defecation households	% increase in open defecation households
**2005**	26,056	8,443	32.40	-
**2006**	26,736	9,207	34.44	2.04
**2007**	27,450	9,711	35.38	0.94
**2008**	28,664	10,473	36.54	1.16
**2009**	28,916	11,127	38.48	1.94
**2010**	30,143	11,606	38.50	0.02
**2011**	31,835	12,325	38.72	0.22
**2012**	32,501	13,163	40.50	1.78
**2013**	33,239	13,733	41.32	0.82
**2014**	33,179	14,432	43.50	2.18
**2015**	32,926	14,422	43.80	0.30
**2016**	31,571	13,958	44.21	0.41

### Data collection

A structured questionnaire consisting of close-ended questions was used for the data collection. The questionnaire asked respondents, “what kind of toilet facility does your household have?” Responses included flush latrine/water closet (WC), ventilated improved pit (VIP/KVIP), other pit latrines, open fields, defecate in the house, faeces transferred elsewhere (bucket latrine) or others. ‘Open fields' response was used for OD. Trained fieldworkers administered questionnaires, and interviews were conducted in privacy at the respondent's place of residence. Interviews were done one-on-one with the respondents in the local language, mainly Twi, widely spoken in Ghana.

### Data management and analyses

Field supervisors checked all the forms manually for completeness and consistency. Range and consistency checks were performed, and the forms were double entered on computers using Microsoft visual FoxPro (version 9.0) data management software. Discrepancies were resolved by referencing the original forms and field manual used to train the data collectors. Data analyses were performed using STATA version 14.

In an exploratory analysis, the correlation (*r*) between population growth (using total number of households as a proxy for population), year, and the population of OD households was obtained. A time series plot from 2005 to 2016 for the total household population, and total households that practice OD was provided. The 12 years (2005 – 2016) average percentage yearly increase in OD was computed and used to crudely project the percentage of OD for the years 2020, 2025 and 2030. Similarly, using the minimum yearly increase in OD over the 12-year period, an estimate was obtained for 2020, 2025 and 2030. In addition, the spatial distribution of OD households for the year 2016 was mapped by sub-district using ArcView Geographic Information Systems software (version 3.1).

### Limitations

The sample size for this study is the number of households for each year, as shown in Table 1 is quite large, but its findings are unlikely to be generalized since the KHDSS covers only two districts out of the 254 districts in Ghana. However, the findings would be quite applicable to districts with similar settings as those used for this study.

### Ethical consideration

Written informed consent was obtained from adult participants involved in interviews as part of the Kintampo Health and Demographic Surveillance activities. The Kintampo Health Research Centre Institutional Ethics Committee reviewed the protocol and all instruments associated with this study as part of the activities of the Kintampo HDSS. The approval certificate details are Ref: KHRC/IEC/ICF/2010-1; FWA: 00011103; and IOR0004854.

## Results

### Population growth, year and open-defecation

**Figure 1:**
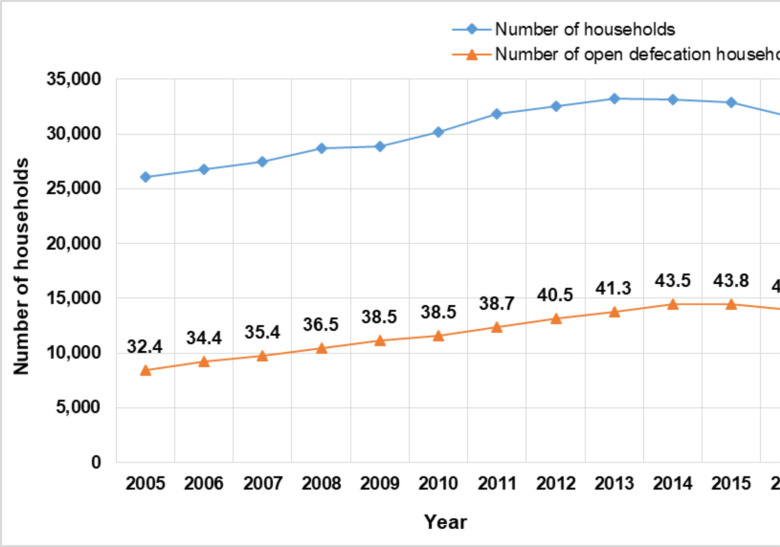
Trend in number of households and open-defecation households in Kintampo North Municipal and Kintampo South District from 2005 to 2016. (The numbers on the plot represent the percentage of household that practice OD).

From 2005 to 2016, the total number of households in the Kintampo area ranged from 26,056 (in 2005) to 33,179 (in 2014). Of these households, ranging from 8,443 to 14,432 practiced OD. For each of the years, the total percentage of household that practiced OD is presented in Figure 1. In the baseline line year (2005), 32.4 percent of the households in the area defecated in the open. This increased to 44.2 percent in 2016.

There was a significant positive correlation between the population (number of households) and OD (*r* = 0.97, *p*-value < 0.001). Likewise, a significant linear association (*ρ* = 0.98, *p*-value < 0.001) was found between year and OD=0.9797 (p-value<0.001).

### Annual change in open-defecation households

The annual percentage change in the proportion of OD households is presented in Table 1. The minimum and maximum yearly percentage increase were 0.02 per cent (from 2009 to 2010) and 2.18 per cent (from 2013 to 2014), respectively (Table 1). The 12-year (2005–2016) average percentage increase in OD households was estimated to be 1.07 per cent.

**Figure 2:**
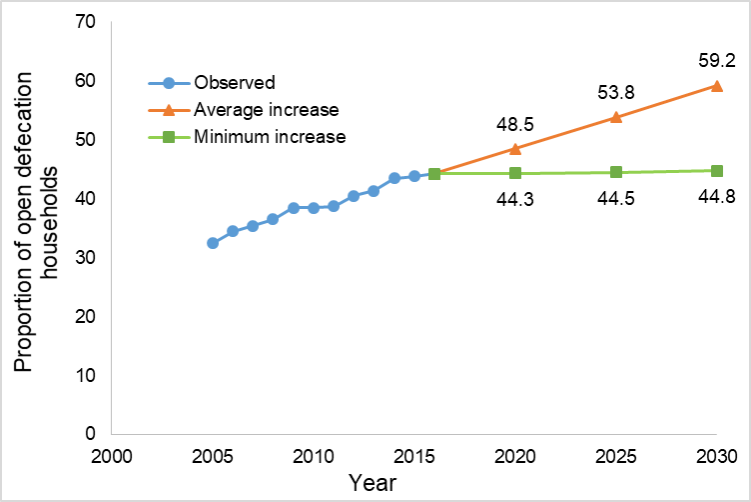
Percentage of household that practice open defecation in Kintampo North Municipal and Kintampo South District from 2005 to 2016, with projection based on minimum and average percentage yearly increases.

All things being equal, if for each year, the proportion of open defecation households increase by the estimated average increase of 1.07 per cent, then by the year 2030, about one in every two households in the study area may be defecating in the open (Figure 2). Similarly, if all things remain the same and the proportion of OD households increase per year by the minimum increase of 0.02 per cent, then by the year 2030, about 45 per cent of the households may be openly defecating (Figure 2).

**Figure 3:**
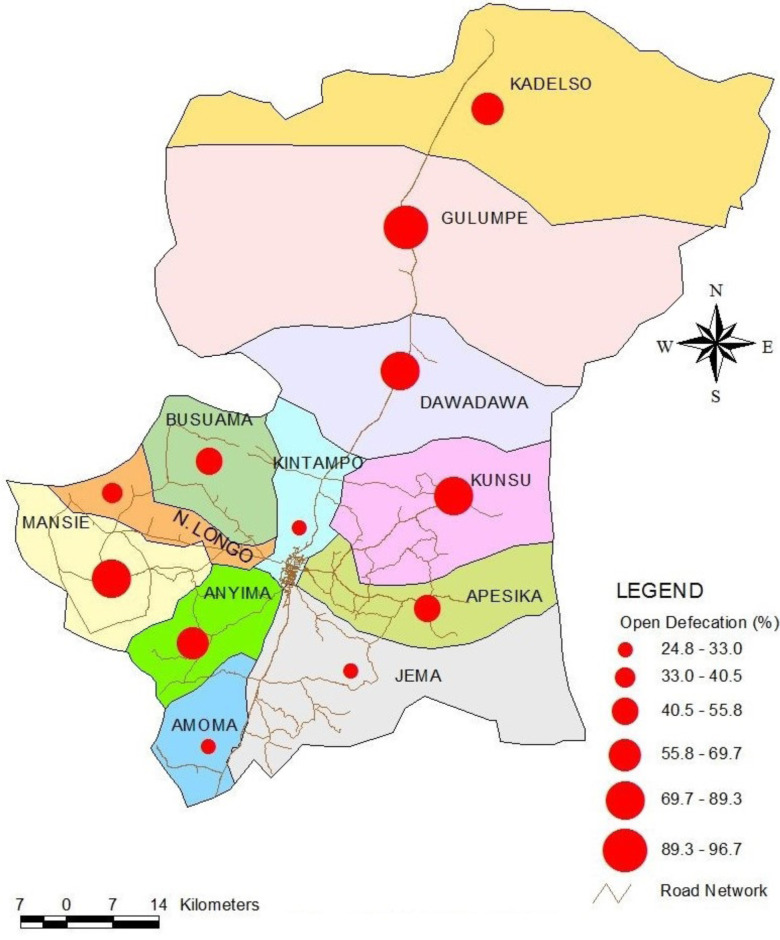
Spatial distribution of open-defecation households in Kintampo North Municipal and Kintampo South District in 2016 by sub-District.

From Figure 3, in six out of the 12 sub-Districts, more than half of the households openly defecate. Four out of these six sub-Districts were located in the Kintampo North Municipal.

## Discussion

This paper highlights the importance and capacity of health and demographic surveillance system for monitoring and evaluating health indices and targets of the sustainable development goals. From the study's findings, it is observed that although SDG target 6.2 aims to end OD by the year 2030, the target of OD free is not achievable in the two districts by the target year if the current trend should continue.

There is a need for the study districts to reverse the OD trend. About 40 per cent of households in Kintampo North Municipal and Kintampo South District used OD. From the trend, the level of OD is expected to continue or even increase if the current trend persists. This argument is supported by the fact that 32.4 per cent of households in the area defecated in the open in 2005, but the trend increased steadily to 44.2 per cent in 2016. This observation suggests that in 2016, more than two households out of 5 defecated in the open within the study area.

The study's findings are not very different from other studies in LMICs 11, 17 or elsewhere in Ghana. Osumanu et al., in a study on OD in the Wa Municipality, found that about 50 per cent of the households had no toilet facility at home, and therefore used OD since unwillingness to pay a fee and other factors deter them from using public latrines.^10^

The results of the projection (Figure 2) show that about 50 per cent of households in the study area may be defecating in the open by the year 2030, which is the exact proportion reported by Osumanu et al. 2019 in the Wa Municipality. The present study observed that, all things being equal, if the proportion of OD households increases by the estimated average increase of 1.07 per cent for each year, about one in two households will be practising OD by 2030. The proportion drops slightly to about 45 per cent of OD households by 2030 if the minimum increase of 0.02 per cent is used for the projection.

According to the findings of this present study, stopping OD is not achievable by 2030 or even beyond if nothing is done to reverse the observed trend in the study area. This is because there was a significant positive correlation between the population/households and OD (*r* = 0.97, *p*-value < 0.001). This implies that the number of OD households increased with the increasing population. Likewise, a significant linear association (*r* = 0.98, *p*-value < 0.001) was found between year and OD. That is, the total number of OD households increased over time. The two districts are largely rural with about a third of the population living in peri-urban settings which are mainly the district capitals. Although a section of the available literature suggests that OD is predominantly a rural phenomenon,^18^–^20^ observations of the present study suggest that OD is more prevalent in the Kintampo North Municipal which is more urban compared to the Kintampo South District. It was observed from the present study that in six out of the 12 sub-Districts, more than half of the households defecate openly, with four out of these six sub-Districts located in the Kintampo North Municipal.

## Conclusion

Evidence from this study indicates that the goal of opendefecation free target by 2030 may not be achieved in the Kintampo North Municipal and Kintampo South District of Ghana if the current trend continues.
